# The Role of AXL Signaling and Mutant *Isocitrate Dehydrogenase 1/2* in Conventional Chondrosarcoma

**DOI:** 10.3390/cancers18121929

**Published:** 2026-06-13

**Authors:** Matthew Chu, Zacharias Barron, Sila Basbay, Kurt Richard Weiss, Karen Schoedel, Ines Lohse

**Affiliations:** 1 Department of Orthopaedic Surgery, University of Pittsburgh School of Medicine, Pittsburgh, PA 15213, USAsib31@pitt.edu (S.B.); krw13@pitt.edu (K.R.W.); 2 UPMC Hillman Cancer Center Academy, University of Pittsburgh, Pittsburgh, PA 15232, USA; 3 School of Medicine, University of Pittsburgh, Pittsburgh, PA 15213, USA; 4 Hillman Cancer Center, University of Pittsburgh, Pittsburgh, PA 15232, USA; 5 Orland Bethel Musculoskeletal Research Center, Department of Orthopaedic Surgery, University of Pittsburgh, Pittsburgh, PA 15213, USA; 6 Department of Pathology, University of Pittsburgh School of Medicine, Pittsburgh, PA 15213, USA

**Keywords:** chondrosarcoma, *IDH1*, *IDH2*, *AXL*, 2-hydroxyglutarate, epigenetics, targeted therapy, bone tumor

## Abstract

Conventional chondrosarcoma (CS), the second most common primary bone malignancy, presents a significant therapeutic challenge due to high levels of resistance to chemotherapy and radiotherapy. Current treatment is limited to surgical resection, which is often incomplete due to tumor involvement of critical structures. Recent molecular profiling studies have highlighted frequent *Isocitrate Dehydrogenase 1* (*IDH1*) and *Isocitrate Dehydrogenase 2* (*IDH2*) mutations, along with AXL phosphorylation and AXL-associated pathway activity, as candidate molecular features in CS. However, their functional roles may vary by subtype and require context-specific interpretation. *IDH1/2* mutations are thought to contribute to CS tumorigenesis through metabolic and epigenetic mechanisms, including D-2-hydroxyglutarate (D-2-HG) accumulation, altered differentiation programs, and epigenetic dysregulation, although direct mechanistic evidence in CS remains less complete than in other *IDH*-mutant malignancies. Concurrently, AXL, which has been implicated in epithelial-to-mesenchymal transition (EMT), immune evasion, and therapeutic resistance, is emerging as a candidate signaling node in CS biology. Potential convergence between *IDH1/2*-associated metabolic or epigenetic states and AXL-associated signaling remains hypothesis-generating and requires CS-specific validation. This review synthesizes current evidence on the roles of *IDH1/2* mutations and dysregulation of AXL signaling in CS, emphasizing their potential contributions to tumor aggressiveness, immune suppression, and resistance to therapy. Additionally, we explore current developments in targeted therapy exploiting *IDH1/2* and *AXL* dysregulation.

## Simple Summary

Conventional chondrosarcoma, although the second most common bone cancer, remains a challenging disease due to high levels of treatment resistance and an aggressive tumor phenotype. Although not for a lack of trying, treatment for most patients is limited to surgical resection. Mutations in Isocitrate Dehydrogenase are frequently observed in conventional chondrosarcoma and have been associated with an aggressive phenotype. Additionally, recent studies have suggested activation of the receptor tyrosine kinase AXL as a potential molecular feature that may contribute to CS pathogenesis and progression. This review explores potential interactions between Isocitrate Dehydrogenase mutation and AXL activation and reviews developments in Isocitrate Dehydrogenase and AXL targeted therapy development.

## 1. Introduction

Conventional chondrosarcoma (CS) represents the second most common primary bone sarcoma and produces cartilage matrix [1]. They are generally slow-growing tumors that infiltrate bone and soft tissue. Grade is an important prognostic factor, and the criteria established by Evans et al. are still applied today [2]. While grade I displays low metastatic potential and an overall 10-year survival rate of 88–95%, grade II and III tumors are associated with increased metastatic potential, 10–30% and 32–71% respectively, and a 10-year survival of 50–70% depending on anatomical location. Dedifferentiated CS has a dismal prognosis, with a 5-year overall survival of only 7–24% [3]. Due to high levels of chemo- and radio-resistance, treatment is centered around surgery with adjuvant radiation and chemotherapy in selected cases [4,5,6,7]. Chemotherapy has demonstrated limited benefits in most CS subtypes, particularly in conventional CS, with similar resistance observed in response to radiotherapy [5,6].

Systemic treatment options for unresectable, recurrent, or metastatic CS remain limited, particularly for conventional CS, although selected subtypes and clinical settings may warrant chemotherapy, radiotherapy, or palliative approaches [4,5,6,7]. Although conventional CS is relatively radioresistant, radiotherapy, including proton or particle therapy, may be considered in selected settings such as skull base, axial, unresectable, incompletely resected, or for palliative disease where local control is difficult, and dose delivery near critical structures is important [8,9]. These approaches should be framed as selected clinical local-control strategies that require validation rather than representing broadly effective systemic or curative treatments for all CS [8,9]. Ozekibart (INBRX-109) has generated interest as an investigational death receptor 5 (DR5) agonist, but its clinical role remains under evaluation and should not be described as an established clinical benefit [10].

This overall adjuvant therapeutic failure suggests that the biological drivers of disease progression and resistance remain incompletely understood. *Isocitrate Dehydrogenase 1/2* (*IDH1/2*) mutations are most characteristic of central and periosteal cartilaginous tumors and can be detected in enchondromas, whereas peripheral CS arising from osteochondromas follow distinct *EXT1/EXT2*-associated biology [11]. Although *IDH1/2* mutations are frequent early events in central/periosteal cartilaginous tumors and are present in some enchondromas, their role in progression and prognosis remains incompletely understood [11].

A second potential signaling node is AXL, which has been implicated in survival, migration, and therapy resistance across several cancers and has been discussed as a potential therapeutic axis in primary bone tumors, although CS-specific evidence remains limited [12].

Because CS comprises biologically distinct entities, the present manuscript focuses primarily on central and periosteal conventional CS and dedifferentiated CS, where relevant to *IDH1/2*, AXL, and progression biology [1,11,13,14]. Peripheral CS arising from osteochondroma follows a distinct *EXT1/EXT2*-associated pathway and does not share the same *IDH1/2*-mutant biology [15,16,17]. Findings should not be generalized to peripheral CS, clear cell CS, mesenchymal CS, or other nonconventional subtypes without subtype-specific evidence [1,15,16,17]. Specific emphasis will be given to the roles of *AXL* and *IDH* as potential biomarkers and emerging targeted therapies capitalizing on *IDH* and *AXL* dysregulation.

## 2. AXL

The AXL Receptor tyrosine kinase is a member of the TYRO3/AXL/MER (TAM) family of receptor tyrosine kinases (RTKs) and is primarily activated by growth arrest-specific 6 (GAS6) [18]. Ligand-dependent AXL autophosphorylation activates downstream phosphoinositide 3-kinase/protein kinase B/mammalian target of rapamycin (PI3K/AKT/mTOR) and Yes-associated protein 1/transcriptional co-activator with PDZ-binding motif (YAP1/TAZ) signaling, pathways that regulate cell survival, motility, and invasion [12,18]. In non-CS tumors, AXL has been associated with invasive phenotypes, therapy-induced plasticity, therapeutic resistance, and poor clinical outcomes [6,12,18,19].

**Figure 1:**
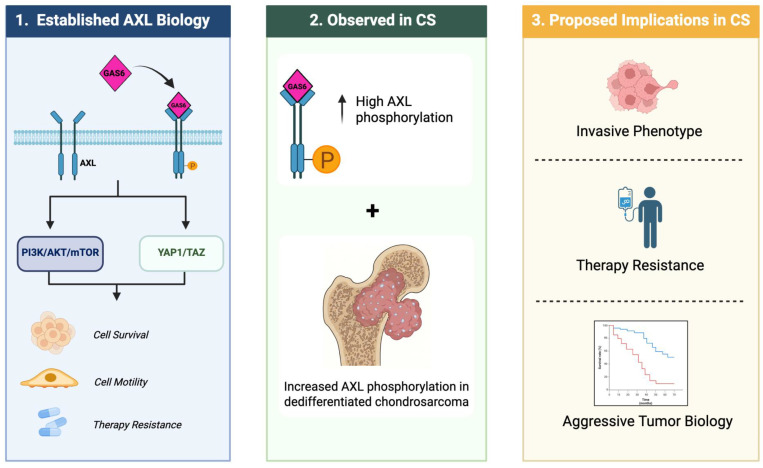
Candidate AXL-signaling in chondrosarcoma. GAS6 binding activates AXL signaling and downstream PI3K/AKT/mTOR and YAP1/TAZ pathways, which are associated with cell survival, motility, and therapy resistance. In chondrosarcoma (CS), increased AXL phosphorylation has been observed in tumor specimens and appears higher in dedifferentiated CS. However, current evidence in CS remains largely correlative, and the exact functional role of AXL signaling in CS progression has not yet been fully established. Together, these findings support a possible role for AXL-associated signaling in aggressive tumor behavior and treatment resistance in CS. Created in https://BioRender.com.

In CS, available evidence supports AXL phosphorylation as a candidate marker of RTK pathway activity in some tumors, but it does not yet establish AXL as an independent driver of CS progression (Figure 1). Zhang et al. performed functional RTK profiling in human CS specimens and identified AXL as one of the most highly phosphorylated RTKs in human tumor specimens, suggesting a correlation between AXL signaling and an aggressive tumor phenotype, supporting AXL as a candidate signaling node [20]. However, this evidence remains primarily correlative and requires functional validation before AXL can be considered a driver in CS. Reviews of CS molecular pathology have discussed potential convergence between RTK activation and PI3K/AKT/mTOR signaling, but this remains a pathway-level synthesis rather than definitive proof of AXL dependency in CS [5]. These findings collectively support the hypothesis that AXL-associated pathway activation may participate in CS progression, although direct functional dependency remains insufficiently established [5,12,18,20].

Furthermore, Zhang et al. identified high levels of AXL phosphorylation in dedifferentiated CS, suggesting that AXL activity may be related to aggressive CS biology, reinforcing the importance of measuring functional AXL activity rather than expression alone [20]. Src-family kinase signaling has also been explored in CS progression, chemoresistance, and migration. Preclinical work suggests that Src-family kinases may contribute to chemoresistance and migration in CS models, and that dasatinib may sensitize selected *TP53*-mutant CS cells to doxorubicin [21]. These findings support interpreting AXL within a broader kinase-network framework rather than as an isolated signaling axis; however, this still needs to be verified with further clinical evidence [12,21]. Collectively, current evidence from multiple tumor systems supports a model in which AXL may function as a dynamically regulated signaling node involved in kinase activity, growth factor signaling, and therapy-associated resistance, while its precise functional role in CS remains incompletely defined [5,6,12,18,19,20].

At present, available CS-specific AXL data are insufficient to determine whether AXL activation varies systematically by anatomic site, grade, or low-metastatic-risk locations [20]. Future studies should therefore stratify *AXL* expression and AXL phosphorylation by grade, histologic subtype, and anatomical site before assigning prognostic significance [1,20].

### Evidence of AXL in Non-CS Bone Sarcoma Models

*Protein Kinase C Iota (PRKCI)* silencing has been reported to suppress downstream AKT/mTOR activation in osteosarcoma (OS) models [22].

Although this may support a possible regulatory relationship between *PRKCI*/GAS6/AXL in primary bone tumor biology, it should not be taken as direct evidence that *PRKCI* regulates AXL-driven proliferation in CS without validation in CS-specific models [22].

Cross-tumor and primary bone cancer literature suggests potential convergence among AXL, insulin-like growth factor/insulin receptor (IGF/IR), PI3K/AKT/mTOR, and YAP1/TAZ signaling; however, the extent to which these interactions operate specifically in CS remains incompletely defined [12].

Furthermore, in colorectal cancer, Dunne et al. identified AXL as a regulator of inherent and chemotherapy-induced invasion and reported that *AXL* expression predicted poor clinical outcome in early-stage colon cancer [19]. Although these findings support the broader role of AXL in invasion and therapy-associated tumor plasticity, they are derived from a non-chondrosarcoma malignancy and should therefore be interpreted only as a supportive biological context for CS.

## 3. *IDH*

Importantly, the molecular framework discussed here applies primarily to central and periosteal CS. Peripheral chondrosarcoma arising from a preexisting osteochondroma follows a distinct *EXT1/EXT2*-associated pathway and should not be assumed to share the same *IDH*-mutant biology as central/periosteal CS [15,16,17].

**Figure 2:**
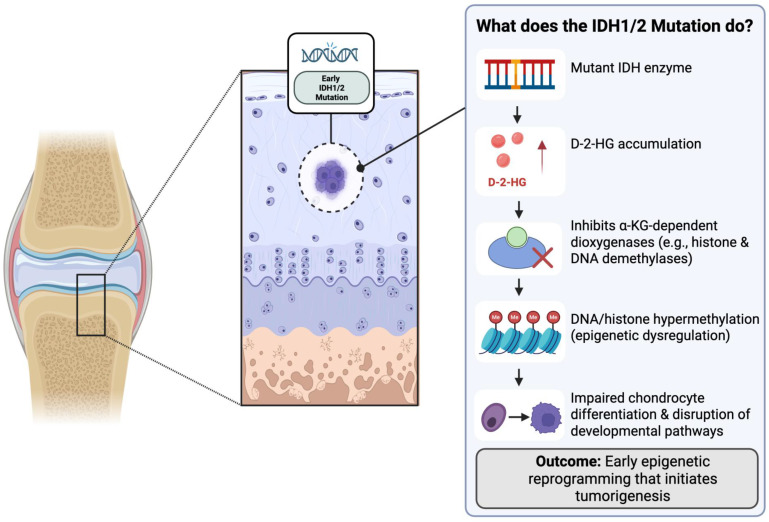
Proposed effects of IDH1/2 mutation in conventional chondrosarcoma (CS). IDH1/2 mutation is depicted as an early event in cartilaginous tissue that results in the accumulation of D-2-hydroxyglutarate (D-2-HG), inhibition of α-KG-dependent dioxygenases, and widespread DNA/histone hypermethylation. These epigenetic changes are proposed to impair chondrocyte differentiation and disrupt developmental pathways, contributing to early tumorigenesis in CS. Created in https://BioRender.com.

Mutations in *IDH1* and *IDH2* are characteristic high-frequency alterations in central and periosteal cartilaginous tumors, including many CS, but they are not universal across all subtypes [11,15,16,17]. *IDH1* or *IDH2* mutations have been reported in approximately 56% of central and periosteal cartilaginous tumors, including CS, while the mutations were rare in other mesenchymal tumors, supporting a lineage-specific role [11]. Subsequently, a meta-analysis of over 400 CS patients estimated *IDH* mutations in approximately half of CS patients, indicating a high-frequency of alteration in CS cohorts [23]. Foundational studies in *IDH*-mutant cancers demonstrated that mutant *IDH* acquires a neomorphic activity that converts α-ketoglutarate (α-KG) to D-2-hydroxyglutarate (D-2-HG); however, evidence is limited in CS (Figure 2) [24].

Mechanistic studies outside of CS showed that D-2-HG competitively inhibits α-KG–dependent dioxygenases, including histone and DNA demethylases [25]. This inhibition results in widespread DNA and histone hypermethylation that is associated with impaired differentiation, yet limited information surrounds this effect in CS. Pathmanapan et al. identified distinct metabolic signatures in *IDH*-mutant CS compared to wild-type tumors [26].

In CS cell lines, mutant IDH inhibition decreases D-2-HG levels, confirming on-target metabolic effects; however, this does not necessarily translate into reversal of tumorigenic phenotypes, suggesting that additional pathways sustain malignant behavior [27].

Rather than primarily increasing proliferation, several studies indicate that mutant *IDH* primarily alters cellular differentiation, although direct CS-specific mechanistic proof remains incomplete. *IDH*-mediated epigenetic changes have been proposed to disrupt normal endochondral ossification and maintain chondrocytes in an immature state that promotes tumor formation [3]. Aberrant DNA methylation has been reported in CS and is biologically consistent with altered epigenetic regulation in *IDH*-mutant tumors, although methylation patterns may also reflect additional subtype- and progression-associated changes [28]. These findings support a model in which *IDH1/2* mutations may contribute to early epigenetic and differentiation changes, although additional genetic and microenvironmental events are required for progression [3,28].

*IDH1/2* mutations support a model in which early metabolic and epigenetic reprogramming contribute to cartilaginous tumor formation in many central and periosteal tumors, but this model should not be interpreted as a universal or linear progression model for all CS subtypes [11,13,14,15,16,17]. No study to date has definitively established a direct mechanistic pathway by which *IDH1/2* mutation induces AXL activation in CS. Current evidence instead consists of separate observations that *IDH1/2*-mutant and wild-type CS display distinct metabolic profiles and that AXL phosphorylation can be detected in human CS specimens [20,26].

Cross et al. support that *IDH1/2* mutation often occurs early, while alterations such as *telomerase reverse transcriptase* (*TERT)* promoter mutation are associated with progression and outcome [14]. However, this model should not be generalized to all CS subtypes or interpreted as a universal linear sequence. Building on, Dermawan et al. further reported that dedifferentiated CS displays distinct methylation patterns and enrichment of *TP53* and *TERT* alterations [13]. Together, these findings support a multi-hit progression model in which dedifferentiated CS is not simply a more aggressive extension of *IDH*-mutant biology, but often reflects additional progression-associated alterations, including *TP53* pathway disruption, *TERT* dysregulation, and *Cyclin-Dependent Kinase Inhibitor 2A* (*CDKN2A)*/p16 loss [13,14].

The relationship between *TERT* promoter alteration and telomerase biology in CS is complex. Earlier studies reported that most chondrosarcomas lacked detectable telomerase activity [29]. By contrast, later genomic studies identified *TERT* promoter mutations as progression-associated events in central CS [14]. Therefore, *TERT* dysregulation should be discussed as part of telomere-maintenance and progression biology rather than equated simplistically with uniformly detectable telomerase activity across CS [14,29].

Because *TP53* pathway disruption and *CDKN2A*/p16 loss are associated with progression and dedifferentiation, AXL activation should not be interpreted in isolation but rather in the context of these cooperating alterations [13,14,30]. Accordingly, *IDH1/2* mutations may be viewed as early lineage-associated events in many central/periosteal cartilaginous tumors, whereas malignant progression, dedifferentiation, and metastasis likely depend on cooperating alterations and may also occur through *IDH*-wildtype evolutionary trajectories.

*IDH*-wildtype CS should also be explicitly considered. Evolutionary studies of central CS indicate that *IDH* status alone does not define all progression trajectories [14]. Recent work further suggests that *IDH1/2* alterations may be absent or even lost during tumor evolution in some metastatic central CS cases [31]. These observations reinforce that CS progression cannot be modeled as a single linear *IDH1/2*-mutant pathway [14,31].

The CS tumor microenvironment has been shown to vary in composition and phenotype, particularly in high-grade and dedifferentiated tumors. Using immunohistochemical profiling, Richert et al. examined 57 surgically resected CS specimens, including conventional and dedifferentiated tumors, and identified a macrophage-dominant immune infiltrate, particularly in high-grade tumors [32]. Providing further support, Kostine et al. performed an analysis of 49 resected CS samples and identified program death ligand 1 (PD-L1) expression on tumor cells together with CD8^+^ cytotoxic T-cell infiltration [33]. The coexistence of tumor-infiltrating cytotoxic T-cells with PD-L1 expression suggests a potential rationale for exploring immune checkpoint-based approaches in selected dedifferentiated CS cases [33]. These studies demonstrate that CS, particularly high-grade and dedifferentiated tumors, can contain immunosuppressive microenvironmental features [32]. However, whether these immune patterns are directly driven by *IDH1/2* mutation status remains unresolved.

Reviews of CS biology, integrating data from human tumor specimens and experimental models, further show interactions between metabolic alterations and oncogenic signaling patterns, including PI3K/AKT/mTOR [5,6]. Mutant *IDH* generates the oncometabolite 2-HG, which inhibits a-ketoglutarate-dependent dioxygenases and produces widespread epigenetic dysregulation.

In broader *IDH*-mutant cancer contexts, epigenetic dysregulation can alter the expression of growth factor receptors and survival pathways; however, the extent to which this directly drives PI3K/AKT/mTOR activation in CS remains incompletely established [5,6,25]. Because PI3K/AKT/mTOR signaling promotes cell survival, proliferation, and resistance to apoptosis in many tumor contexts, epigenetic and metabolic changes in CS may plausibly intersect with survival pathways; however, direct *IDH*-driven activation of this axis in CS remains incompletely established as well [5,6,25].

### 3.1. Interactions Between IDH1/2 Mutations and AXL Signaling in CS

No study to date has definitely established a direct mechanistic pathway by which *IDH1/2* mutation induces AXL activation in CS. Current evidence instead consists of separate observations that *IDH1/2*-mutant and wild-type CS display distinct metabolic profiles and that AXL phosphorylation can be detected in human CS specimens [20,26]. *IDH1/2* mutations have been demonstrated to result in the accumulation of the oncometabolite D-2-hydroxyglutarate (D-2-HG) [24,27].

In other *IDH*-mutant contexts, D-2-HG has been linked to altered hypoxia-related signaling; however, a direct *IDH1/2* to hypoxia-inducible factor-1 alpha (HIF-1α) to GAS6/AXL regulatory axis has not been demonstrated in CS and should therefore be interpreted as extrapolative rather than established CS biology. *IDH*-mutant and *IDH*-wildtype CS display distinct metabolic profiles, including differences in pathways related to glycolysis, glutamine metabolism, and oncometabolite production; however, these observations do not establish that glycolytic or glutamine-related changes directly drive AXL phosphorylation, aggressive behavior, or therapeutic resistance in CS [26]. Whether these metabolic features directly predict aggressive behavior or therapeutic response in CS remains incompletely established.

Aberrant AXL signaling can activate downstream pathways such as PI3K–AKT–mTOR and mitogen-activated protein kinase (MAPK), which play critical roles in cell survival, migration, and immune evasion [5,6,12,18]. The coexistence of frequent *IDH1/2* mutations and aberrant AXL phosphorylation in CS raises a testable possibility of pathway convergence, but current evidence does not prove that IDH-associated metabolic states directly regulate AXL signaling or downstream CS phenotypes [14,20,26]. Future studies may directly compare *AXL* expression, AXL phosphorylation, and downstream pathway activation in *IDH1/2*-mutant versus *IDH1/2*-wildtype CS cohorts using available transcriptomic, proteomic, or phosphoproteomic datasets where associated molecular and clinical data are available [14,20,26].

AXL has also been implicated in glioma growth, migration, and invasion, and experimental inhibition of AXL signaling suppressed glioma growth and invasion in preclinical models [34]. However, glioma biology differs substantially from CS, including the prognostic meaning of *IDH* mutation. Therefore, glioma data may provide useful conceptual context for AXL-associated invasion and resistance, but should not be directly extrapolated to CS without disease-specific validation.

### 3.2. IDH1/2 and AXL as Biomarkers

The prognostic meaning of *IDH1/2* mutation in CS is best interpreted through a multi-factor framework that accounts for tumor grade, histologic subtype, clinical endpoint, *IDH1* versus *IDH2* status, and cooperating genomic alterations rather than through *IDH* status alone [13,14,23,30,35]. Meta-analytic data suggest that the prognostic significance of *IDH1/2* mutations is not uniform across cohorts, reinforcing the need to interpret *IDH* status in relation to grade, histologic subtype, clinical endpoint, and co-alterations such as *TERT*, *TP53*, and *CDKN2A* [23]. The apparent discrepancies across studies may partly reflect differences in cohort composition and outcome structure, because different analyses have evaluated various distinct endpoints, including overall survival, relapse-free survival, metastasis-free survival, and grade-stratified subgroup outcomes [23,30,35].

A useful explanatory framework is that *IDH1/2* mutations may function as early lineage-associated and differentiation-altering events in many central and periosteal cartilaginous tumors, whereas progression, dedifferentiation, metastasis, and therapeutic resistance are likely shaped by additional genomic events [3,11,13,14,30,31]. Under this model, conflicting prognostic data may reflect differences in cohort composition and endpoint selection rather than true contradiction [23,30,35]. Studies enriched for lower-grade or less aggressive central tumors may identify *IDH*-mutant disease with favorable relapse-free or metastasis-free outcomes, whereas cohorts enriched for high-grade, dedifferentiated, *IDH1*-mutant, *TERT*-altered, *TP53*-disrupted, or *CDKN2A*/p16-altered tumors may show worse outcomes [13,14,30,35].

*IDH1* mutations, particularly when co-occurring with *TERT* promoter alterations, have been associated with poorer prognoses and are observed in approximately 20% of CS cases, whereas *IDH2* mutations do not correlate with worse outcomes even in the presence of *TERT* [14]. These findings highlight the distinct biological behavior of *IDH1* versus *IDH2* mutations and underscore the importance of stratifying patients based on specific mutational profiles. Similarly, Zhu et al. performed sequencing of *IDH1/2* in 89 central CS cases and identified *IDH1/2* mutations in 46% of tumors. Although *IDH1/2* mutation status was not associated with overall survival, mutant tumors showed longer relapse-free and metastasis-free survival within the high-grade CS subgroup, supporting the interpretation that the prognostic significance of *IDH1/2* mutations is context-dependent rather than uniformly adverse [30]. Zhu et al. also reported recurrent co-alterations involving *TERT*, *CDKN2A/2B*, and *TP53* in subsets of high-grade and dedifferentiated CS, suggesting that progression may depend on cooperating genomic alterations rather than *IDH1/2* mutation alone [30].

Complementing these findings, Nacev et al. analyzed 2138 sarcomas across 45 histologic subtypes using MSK-IMPACT and demonstrated recurrent *IDH1* mutations alongside *TERT* promoter alterations in CS [36]. However, because this was a broad multi-sarcoma sequencing cohort, CS-specific prognostic conclusions still require subtype- and grade-stratified outcome studies [36].

Patients with *IDH*-mutant atypical cartilaginous tumors (ACT) and grade I CS generally demonstrate favorable outcomes, with high long-term survival and rare metastatic events. *IDH* status should not be interpreted here as the sole determinant of prognosis [3]. In contrast, the prognostic significance of *IDH1/2* mutations in higher-grade CS remains inconsistent across cohorts. In one recent analysis of grade II/III and dedifferentiated CS, *IDH*-wildtype status was associated with longer overall survival than *IDH*-mutant disease, with particularly poor outcomes reported among dedifferentiated *IDH*-mutant tumors [35]. However, this finding should be interpreted alongside other studies reporting different associations between *IDH* status and outcome, including cohorts in which *IDH1/2* mutations were associated with longer relapse-free and metastasis-free survival in high-grade CS [30]. Rather than representing a simple contradiction, these findings suggest that the apparent prognostic direction of *IDH1/2* mutation depends on cohort composition, endpoint selection, and progression-associated co-alterations. *IDH* status alone is therefore insufficient as a universal prognostic marker and should instead be interpreted in relation to grade, histologic subtype, clinical endpoint, *IDH1* versus *IDH2* status, and cooperating alterations such as *TERT*, *TP53*, and *CDKN2A*/p16 [13,14,30,35].

In this context, AXL activation may act as a potential candidate biomarker of aggressive signaling in selected CS cases, but its prognostic value has yet to be validated in sufficient CS cohorts. Together, these findings may suggest that *IDH* status helps define prognostic subsets only when interpreted alongside tumor grade, histologic subtype, clinical endpoint, *IDH1* versus *IDH2* status, and cooperating alterations such as *TERT*, *TP53*, and *CDKN2A*/p16, while AXL phosphorylation may represent a candidate marker of RTK pathway activity in selected CS cases, but whether it provides prognostic information beyond established clinicopathologic and genomic variables remains unvalidated [13,14,20,30,35].

### 3.3. Therapeutic Targeting of IDH1/2 and AXL in CS

While surgery with negative margins is the foundation of treatment in most CS patients, chemotherapy and/or radiation therapy can be added in selected settings. Responses are limited clinically due to high levels of chemo- and radio-resistance, and effective systemic options remain limited for unresectable, recurrent, or metastatic disease. This therapeutic gap has driven the investigation of molecularly targeted and immune-based approaches in advanced CS [5,6,7]. Recent genomic characterization studies have expanded the list of candidate therapeutic targets in CS, and molecularly guided systemic therapy remains investigational for most patients [37]. Beyond IDH inhibition, candidate investigational strategies in advanced CS include kinase-pathway inhibition, immune-based approaches in selected dedifferentiated tumors, and epigenetic therapy, but none have become a validated standard for most patients with advanced CS [6,7,32,33,38]. The clinical relevance of *IDH1* mutations has been evaluated in a phase I trial of the selective IDH1 inhibitor ivosidenib in 21 patients with advanced *IDH1*-mutant CS, including 13 conventional CS cases. Patients received daily oral doses of 500 mg, which were well tolerated with no treatment-related deaths and only one grade 3 adverse event. Treatment resulted in marked reductions in plasma 2-hydroxyglutarate to levels comparable to those of healthy individuals, confirming on-target metabolic inhibition [4]. Clinically, ivosidenib was associated primarily with disease stabilization rather than objective tumor regression. Although median progression-free survival was modest, a subset of patients experienced prolonged disease control, indicating a heterogeneous response distribution rather than uniform benefit [4].

The clinical experience with ivosidenib highlights an important therapeutic disconnect that on-target suppression of D-2-HG does not necessarily translate into objective tumor regression in CS [4]. This may reflect the role of *IDH1/2* mutation as an early metabolic and differentiation-altering event, while established malignant behavior may be sustained by cooperating alterations, persistent epigenetic states, or bypass signaling [3,13,14,27,30,39]. Consistent with this possibility, mutant IDH1 inhibition can reduce D-2-HG levels in CS cell lines without consistently reversing tumorigenic properties [27]. Future studies should therefore evaluate mechanisms of limited response or resistance to IDH-directed therapy.

Because *IDH1/2*-mutant CS can exhibit epigenetic dysregulation, epigenetic therapies, including DNA methyltransferase and histone deacetylase inhibitors, have been proposed as investigational strategies. However, these approaches remain preclinical or early-stage in CS and should not be interpreted as established therapeutic options [28,38,39].

Together, these findings support mutant IDH as a pharmacologically targetable alteration in CS, but they also indicate that on-target metabolic inhibition is not sufficient to produce consistent objective tumor regression, highlighting the need to define intrinsic and acquired resistance mechanisms in the future [4,27,39].

Collectively, current studies suggest that *IDH* mutations function as early differentiation-disrupting events, whereas genomic alterations and microenvironmental changes act as downstream drivers to tumor progression [3,11,13,14,23,36].

AXL-directed therapy remains far less developed in CS than IDH-directed therapy. Several AXL-targeting strategies, including selective AXL inhibitors, have been evaluated across other non-CS solid tumor settings, but CS-specific clinical evidence remains lacking [40]. This gap likely reflects the limited amount of CS-specific AXL functional data, the absence of prospectively validated biomarkers, and the lack of clinical trials stratifying CS patients by AXL phosphorylation or pathway activation [20,40]. Therefore, AXL inhibition in CS should currently be viewed as a hypothesis-generating strategy rather than an established treatment approach [20,40]. Future studies should prioritize patient selection based on AXL phosphorylation, pathway activation, and co-alterations that may identify tumors more likely to depend on AXL-associated signaling [20].

The clinical role of epigenetic therapy in CS remains undefined, and available evidence does not establish DNA methyltransferase or histone deacetylase inhibition as an effective standard treatment. CS remains therapeutically challenging because of histologic and molecular heterogeneity, limited sensitivity to conventional systemic therapy, and the lack of validated targeted treatments for most patients with advanced disease. Emerging molecularly guided approaches are promising but remain investigational and require subtype-specific validation [7].

Taken together, *IDH1/2* mutation and AXL-associated signaling remain biologically relevant but incompletely validated therapeutic concepts in CS. Their clinical interpretation requires attention to subtype, grade, endpoint, and co-alterations, and combined IDH/AXL-directed strategies remain untested in CS models or clinical trials [12,13,14,20,23,30,35,40]. CS-specific AXL data remain limited, with much of the therapeutic rationale extrapolated from other broader solid tumor or primary bone cancer literature [12,18,20,40]. Finally, combined IDH/AXL targeting has not yet been validated in CS models or clinical trials.

## 4. Critical Appraisal and Future Directions

In all, the available literature supports biological relevance for *IDH1/2* mutation and AXL-associated signaling in CS, but several limitations soften direct interpretation. *IDH1/2* mutations appear most strongly supported as early metabolic and differentiation-altering events in many central and periosteal cartilaginous tumors, whereas progression, dedifferentiation, and metastatic behavior likely require additional genomic events [3,11,13,14,30,31]. Cs-specific AXL evidence still remains limited and is strongest for observed AXL phosphorylation and pathway-level plausibility rather than direct proof for AXL dependency [12,20,40]. Proposed links among *IDH1/2* mutation, glycolysis, gluatbilimine metabolism, HIF-1α signaling, GAS6/AXL activation, and downstream PI3K/AKT/mTOR or MAPK signaling remain the hypothesis generating [20,26]. Finally, although IDH inhibition demonstrates on-target metabolic activity, clinical benefit mainly takes the form of disease stabilization rather than consistent tumor regression, and mechanisms of limited response or resistance remain incompletely defined [4,27,39]. Future studies should prioritize grade- and subtype-stratified CS cohorts, integrated genomic profiling, functional CS models, and therapeutic studies guided by demonstrated pathway dependence rather than pathway presence alone.

## 5. Conclusions

CS remains a therapeutically challenging malignancy due to high levels of histologic and molecular heterogeneity, limited surgical options in complex anatomical regions, and the lack of validated targeted treatments for most patients with advanced disease. Emerging molecularly guided approaches are promising but remain investigational and require subtype-specific validation [7]. The studies summarized herein highlight IDH1/2 mutations and AXL-associated pathway activity as candidate molecular features relevant to CS biology, prognosis, and therapeutic investigation.

However, much of the mechanistic rationale for AXL in invasion, immune suppression, and resistance derives from non-CS tumor systems, whereas the CS-specific literature is currently strongest for AXL pathway activity as a candidate signal rather than a validated dependency. Direct CS-specific evidence linking mutant *IDH1/2* to AXL activation remains limited, and the prognostic significance of *IDH1/2* mutations varies across CS subtype, grade, endpoint, and co-mutational context [13,14,20,23,30,35].

**Figure 3:**
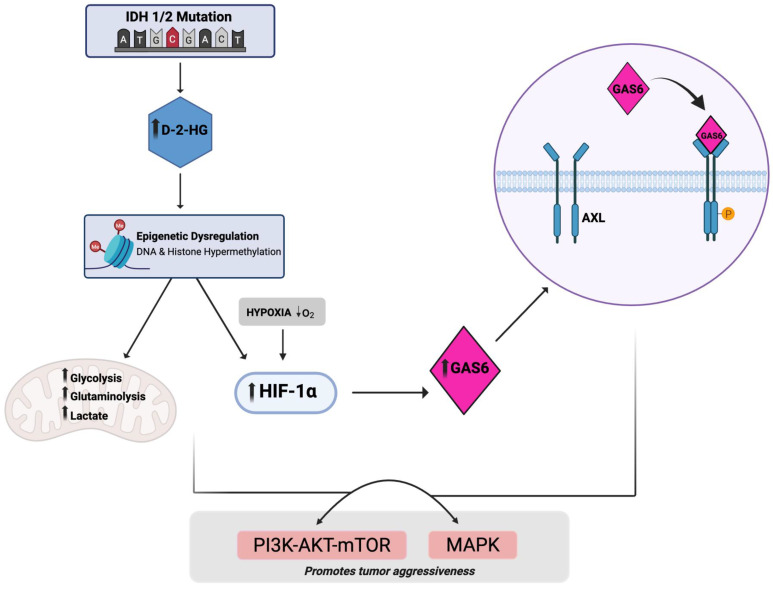
Proposed convergence of IDH1/2 mutations and AXL signaling in chondrosarcoma. IDH1/2 mutations drive the accumulation of D-2-hydroxyglutarate (D-2-HG), leading to epigenetic dysregulation and metabolic reprogramming associated with elevated HIF-1α levels. In parallel, AXL signaling is activated through ligand-dependent engagement by GAS6. Although a direct mechanistic link between IDH-driven metabolic stress and AXL signaling in chondrosarcoma has not been definitively established, hypoxic or metabolically stressed conditions may indirectly enhance AXL signaling. Convergence of IDH- and AXL-associated signaling on shared downstream pathways, including PI3K–AKT–mTOR and MAPK, promotes tumor aggressiveness, therapy resistance, and invasive behavior. Created in https://BioRender.com.

Future research in CS aimed at elucidating the interplay between *IDH*- and *AXL*-mediated signaling, particularly in models of high-grade and dedifferentiated CS (Figure 3), has the potential to uncover novel treatment options for CS patients and increase survival. To this end, determining whether these pathways independently or jointly contribute to aggressive CS phenotypes will be important for future biomarker and therapeutic studies.

## Data Availability

No new data were created or analyzed in this study.

## Abbreviations

Chondrosarcoma (CS), epithelial-to-mesenchymal transition (EMT), protein kinase C iota (*PRKCI*), atypical cartilaginous tumor (ACT), chondrosarcoma (CS), D-2-hydroxyglutarate (D-2-HG), death receptor 5 (DR5), growth arrest-specific 6 (GAS6), hypoxia-inducible factor-1α (HIF-1α), insulin-like growth factor/insulin receptor (IGF/IR), mitogen-activated protein kinase (MAPK), programmed death ligand 1 (PD-L1), phosphoinositide 3-kinase/protein kinase B/mammalian target of rapamycin (PI3K/AKT/mTOR), receptor tyrosine kinase (RTK), TYRO3/AXL/MER (TAM), telomerase reverse transcriptase (TERT), Yes-associated protein 1/transcriptional co-activator with PDZ-binding motif (YAP1/TAZ), *Cyclin-Dependent Kinase Inhibitor 2A* (CDKN2A).
